# Recruitment to diagnosis of urinary tract infections in young children (DUTY) study: an evaluation of the successful methods used in a primary care, prospective cohort study

**DOI:** 10.1186/1745-6215-14-S1-P37

**Published:** 2013-11-29

**Authors:** Cherry-Ann Waldron, Emma Thomas-Jones, Timothy Pickles, Kerry Hood, Kim Harman, Harriet Downing, Kate Martinson, Marilyn Peters, Alastair D Hay, Christopher C Butler

**Affiliations:** 1 South East Wales Trials Unit (SEWTU), School of Medicine, Cardiff University, Cardiff, UK; 2 Centre for Academic Primary Care, School of Social and Community Medicine, University of Bristol, Bristol, UK; 3 Department of Primary Medical Care, University of Southampton, Southampton, UK; 4 King's College London School of Medicine, Division of Health and Social Care Research, Department of Primary Care and Public Health Sciences, London, UK; 5 Institute of Primary Care and Public Health, School of Medicine, Cardiff University, Cardiff, UK

## Introduction and aims

DUTY is a prospective cohort study to derive a clinical algorithm for diagnosis of urinary tract infections in acutely unwell children in primary care.

It provides an example of successful recruitment to a complex paediatric study, requiring the collection of a urine sample from young, unwell children. The aim is to describe and evaluate factors that contributed to its success from a study management and recruiter perspective.

## Method

Four centres (Bristol, Cardiff, Southampton, London) coordinated recruitment from primary care sites across England and Wales. Two recruitment methods were available: 'Option 1' where research nurses supported, and 'Option 2' involving autonomous recruitment by NHS primary care team.

An evaluation of these methods and analysis of feedback questionnaires given to recruiting sites and research nurses involved in DUTY will be made.

## Results

Out of 233 sites, 43% employed option 1 recruitment. 7163 children were recruited and 6390 (89%) urine samples were retrieved. (Bristol=2947 recruits, 92% retrieval rate, Cardiff=1768, 89%, London=1435, 84% and Southampton=1013, 90%).

Further analysis regarding option 1 and option 2 recruitment methods and feedback from recruiting sites and research nurses will be available for presentation in November 2013.

## Conclusion

The success of DUTY to recruit above target is attributed to continuous availability of peer-support, adequate service support reimbursement, real-time data monitoring through web-based data entry, and the flexible offer of two recruitment methods. Feedback from recruiting sites and research nurses provides valuable insight into how recruitment in primary care can be optimised and improved in the future.

